# Role of diabetes health literacy, psychological status, self-care behaviors, and life satisfaction in predicting quality of life in type 2 diabetes

**DOI:** 10.1038/s41598-024-51245-x

**Published:** 2024-01-18

**Authors:** Alireza Jafari, Mahdi Moshki, Mousa Ghelichi-Ghojogh, Mahbobeh Nejatian

**Affiliations:** 1 Department of Health Education and Health Promotion, School of Health, Social Development and Health Promotion Research Center, Gonabad University of Medical Sciences, Gonabad, Iran; 2 Department of Health Education and Health Promotion, School of Health, Social Determinants of Health Research Center, Gonabad University of Medical Sciences, Gonabad, Iran; 3 Neonatal and Children’s Health Research Center, Department of Biostatistics and Epidemiology, Faculty of Health, Golestan University of Medical Sciences, Gorgan, Iran; 4 Social Determinants of Health Research Center, Gonabad University of Medical Sciences, Gonabad, Iran

**Keywords:** Metabolic disorders, Health care, Psychology

## Abstract

One of the factors that can affect the quality of life is the psychological status of people. Also, the psychological status of individuals can be affected by other variables. Therefore, this study was aimed to determine the role of diabetes health literacy, psychological status, self-care behaviors, and life satisfaction in predicting the quality of life in Iranian patients with type 2 diabetes. This cross-sectional study was conducted in Iran in 2021 among 300 patients with type 2 diabetes. The method of selecting the participants in this study was the proportional stratified sampling method. Data collection instruments included six questionnaires of demographic questionnaire and diabetes status, DASS-21 questionnaire (depression, anxiety, and stress), diabetes health literacy scale, self-care behavior questionnaire, satisfaction with life scale (SWLS), and quality of life questionnaire. Statistical methods such as independent sample t test, one-way analysis of variance, linear regression test, and Pearson correlation were used to analyze the data. Based on the results of Pearson correlation test, there was a positive and significant correlation between diabetes health literacy (r = 0.438, p < 0.001), depression (r = − 0.380, p < 0.001), anxiety (r = − 0.355, p < 0.001), stress (r = − 0.423, p < 0.001), and SWLS (r = 0.265, p < 0.001) with quality of life. Based on the results of linear regression, variables of diabetes health literacy, life satisfaction, self -care behaviors, depression, stress, anxiety, age of onset of diabetes and diabetes duration were able to predict 31% of the quality of life (p < 0.001). The results of this study indicated that diabetes health literacy, life satisfaction, stress and the duration of diabetes are able to predict the quality of life of patients with diabetes. Therefore, it is necessary to pay more attention to these effective variables training programs, especially health literacy, the effect of which is very obvious in this study.

## Introduction

Diabetes is one of the most common non-communicable diseases in the world^1^. The prevalence of diabetes in developing countries is much higher than that of developed countries, and it is estimated that by 2040 there will be approximately 642 million type 2 diabetes in the world^2^. The prevalence of diabetes in Iran was reported to be approximately 15% between 2014 and 2020^3^ and will be expected to increase by 2030^4^.

The fundamental goal of diabetes treatment is to improve the quality of life (QOL)^5^. QOL refer to as one's perception of one's position in life according to the value system, culture, standards, expectations, goals, and concerns^6,7^. In fact, diabetes threatens every dimension of QOL (including physical, social and psychological dimensions)^8^ and QOL of diabetic is lower than that of the general population in the community^5,9^. Therefore, identification and assessment of factors affecting QOL in diabetes may be a critical step in improving QOL^10,11^.

Diabetes is associated with an increased risk of mental illness, with depression, anxiety and stress being the most common side effects^12,13^. Depression is common in patients with type 2 diabetes, and it is estimated that patients with type 2 diabetes are approximately twice as likely to suffer from depression as the rest of the community^14^. In addition, depression and anxiety are associated with elevated blood glucose levels in diabetic^12^. In one study, about 14% of people with type 2 diabetes had major depression, 36% had major anxiety, and 24% had severe stress^15^. The results of a study on type 2 diabetes in Iran showed that 46% of patients suffered from depression^16^. Depression in patients with diabetes increases diabetes complications, mortality, and healthcare costs^17^. In fact, comorbidity of type 2 diabetes and mental problems increases the risk of hyperglycemia, cardiovascular disease, complications of diabetes, health care costs, and ultimately decline QOL^18^. Depression and anxiety reduce QOL of people with diabetes^19,20^. There is also a significant statistical relationship between depression and self -care behaviors, with increasing severity of depression associated with decreased self-care behaviors^21^.

Diabetes health literacy is one of the variables that can predict QOL of patients with type 2 diabetes^22,23^. Health literacy is defined as an individual's ability to access, process, and understand healthcare information in order to make appropriate decisions about health conditions^24^. The results of an Iranian study on type 2 diabetes showed that higher diabetes health literacy was associated with higher QOL^8^. The results of the studies have shown that there is a significant relationship between the level of health literacy in diabetic and self -care behaviors, and increasing the level of health literacy can enhance their self -care behaviors^25,26^. Therefore, improving the level of health literacy among people with diabetes and supporting people with depression is crucial to increase their participation in self-care behaviors^27,28^.

Self-care is also one of the general elements of managing diabetes and improving QOL in people with type 2 diabetes^29^. Self-care behaviors in people with diabetes include blood glucose checking, healthy nutrition, medical care, physical activity, problem-solving skills, adaptive and risk-reducing behaviors^30^. The results of a study of Iranian patients with type 2 diabetes showed that people were less able to care for themselves^31^. However, engaging in regular self-care behaviors has a positive relationship with glycemic control, reduced complications, and ultimately improved QOL^32^. Finally, life satisfaction is also lower in people with diabetes^33^. Life satisfaction refers to the attitude of people about generally the one’s life status^34^. There is a correlation between mental problems and life satisfaction in diabetic^35,36^. Furthermore, low life satisfaction in diabetic negatively affects treatment and self-care behaviors^37^.

Overall, it can be concluded that various studies have shown relationships between different variables, such as health literacy, psychological status, self-care behaviors, life satisfaction, and QOL. However, no studies have examined all of these variables and their predictive effects on QOL in people with type 2 diabetes. Therefore, this study aimed to determine the role of diabetes health literacy, psychological status, self-care behaviors, and life satisfaction as predictors of QOL in Iranian patients with type 2 diabetes.

## Method

This cross-sectional study was performed among 300 type 2 diabetes in Gonabad, Iran, in 2021.

### Sample size

Based on a previous study^38^, the sample size was calculated to be 300 participants with a confidence level of 0.95%, a power test of 80%, an accuracy/d of 0.08, and a standard deviation (QOL) of 0.62.

### Sampling method

The method of selecting the participants in this study was the proportional stratified sampling method. The number of health service centers in Gonabad city and the population size of each center were initially determined. Next, each center was considered a stratum and the sample size was determined by the population of each stratum. Finally, the samples from each center were randomly selected from those who had the inclusion criteria for the study. The age over 18 years, residents of Gonabad city, type 2 diabetes diagnosed based on laboratory results, had desire to participate in the study, and be passed over 1 year from the diagnosis of the disease were the inclusion criteria. The questionnaires of people with incomplete information were excluded from the study.

### Data collection instruments

Data collection tools included six instruments of demographic questionnaire and diabetes status, depression, anxiety, and stress questionnaire (DASS-21), diabetes health literacy scale, self-care behavior questionnaire, satisfaction with life scale (SWLS), and quality of life (QOL) questionnaire.

#### Demographic questionnaire and diabetes status

This questionnaire includes questions such as education status, age, employment status, sex, habitation status, marital status, age of onset of diabetes, diabetes duration, history of diabetes in the family, and see diabetic foot complications in others.

#### DASS-21 questionnaire

The questionnaire was developed by Lovibond to measure stress, anxiety, and depression and consists of 21 items^39^. The DASS-21 questionnaire consists of 3 subscales of stress, anxiety, and depression, each consisting of 7 items. In this scale, items are measured using a four-items Likert scale (0 = Did not apply to me at all, 1 = Applied to me to some degree, or some of the time, 2 = Applied to me to a considerable degree, or a good part of time, 3 = Applied to me very much, or most of the time). The final score for each subscale is calculated by summing the items. The total score ranges from zero to 63, with lower scores indicating better psychological status. The validity and reliability of this tool were confirmed in a study in Iran and Cronbach's alpha coefficient of stress, depression, and anxiety was reported 0.78, 0.77, and 0.73, respectively^40^. The validity and reliability of the questionnaire was reviewed in this study and the Cronbach's alpha for all questions of DASS and subscales of depression, anxiety, and stress was calculated 0.948, 0.874, 0.854, and 0.853, respectively.

#### Diabetes health literacy scale

The questionnaire consists of 14 questions and measures three subscales of informational health literacy, numerate health literacy, and communicative health literacy. The questionnaire was designed by Lee, and the validity and reliability of the questionnaire were verified^41^. In Lee's study, Cronbach's alpha of diabetes health literacy and sub-scales of informational health literacy, numerate health literacy, and communicative health literacy were reported 0.90, 0.80, 0.85, and 0.90, respectively^41^. The validity and reliability of this questionnaire was investigated in Iran by Moshki^42^. Cronbach's alpha of total diabetes health literacy scale and sub -scales of numerate health literacy, informational health literacy, and communicative health literacy were 0.919, 0.879, 0.865, and 0.784, respectively. Also, Intra -class correlation of total diabetes health literacy scale and sub -scales of numerate health literacy, informational health literacy, and communicative health literacy were 0.957, 0.921, 0.976, and 0.911, respectively^42^. In this scale, items are measured using a five-items Likert scale (not really = 1 to very much = 5). The total score ranges from 14 to 70, with higher scores indicating better diabetes health literacy status.

#### Self-care behavior questionnaire

The 10-question examines patients' self-care behaviors over the past 7 days, related the diet, exercise, blood glucose testing, foot care and smoking. The questions in this section were measured using a 5-items Likert scale (too much, much, at all, low, very low). The total score ranges from 10 to 50, with higher scores indicating better Self-care behavior status^43^. The validity and reliability of the questionnaire was reviewed in this study and the Cronbach's alpha for all questions was 0.650.

#### SWLS

This scale was designed by Diener^44^. This scale consists of five items that measure the well -being status. In this scale, items are measured using a seven-items Likert scale (completely disagree to completely agree). The total score ranges from 5 to 35, with higher scores indicating better life satisfaction. The validity and reliability of the questionnaire was reviewed in this study and the Cronbach's alpha for all questions was 0.927.

#### QOL questionnaire (SF-12)

The SF-12 questionnaire is a shorter form of SF-36 that survey the QOL and was designed by Ware^45^. This scale consists of 12 questions and 8 subscales of role limitations due to physical problems (RP = 2 items), physical functioning (PF = 2 items), bodily pain (BP = 1 item), general health (GH = 1 item), vitality (VT = 1 item), role limitations due to emotional problems (RE = 2 items), perceived mental health (MH = 2 items), and social functioning (SF = 1 item). These 8 subscales are divided into two components of Mental Health with 6 items (MH, SF, RE, and VT) and Physical Health with 6 items (PF, RP, GH, and BP). The total score ranges from 12 to 47, with higher scores indicating better QOL. The validity and reliability of this questionnaire was investigated in a study in Iran and Cronbach’s alpha for components of Mental Health and Physical Health was reported 0.72 and 0.73, respectively^46^. The validity and reliability of the questionnaire was checked in this study and the Cronbach's alpha for all questions of QOL and components of Physical Health and Mental Health was calculated 0.735, 0.762, and 0.60, respectively.

### Statistical analysis

In this study, SPSS version 22 software was used to analyze the data. To evaluation the relationship between a quantitative variable and three- categorical or more variables, One-way ANOVA was conducted. To evaluation the relationship between a quantitative variable and two- categorical qualitative variables, Independent-samples t-tests was conducted. Linear regression test was performed to evaluation the role of variables in prediction the QOL. To evaluation the correlation between two quantitative variables, Pearson correlation was conducted.

### Ethics approval and consent to participate

This study was based on a research project approved by Ethics Committee of Gonabad University of Medical Sciences with the code of ethics IR.GMU.REC.1400.119. All procedures performed in this study were in accordance with the ethical standards of the institutional and/or national research committee and with the 1964 Helsinki declaration and its later amendments or comparable. Written Informed Consent was obtained from all subjects and/or their legal guardian(s) and for illiterate participants.

## Results

In the present study, most participants were male (59%) and married (91%). The age of onset of diabetes in most people was over 40 years old, and 37.1% reported that more than 10 years have passed since onset of their diabetes. Other demographic information can be seen in Table 1. In this study, the mean (SD) of age, age of onset of diabetes, depression, anxiety, stress, diabetes health literacy, self-care, life satisfaction, and QOL was 58.37 (22.71), 47.04 (10.79), 4.76 (3.87), 4.91 (3.78), 5.56 (3.96), 43.16 (8.99), 30.77 (4.24), 22.47 (5.69), and 32.81 (5.16), respectively.

**Table 1:** Characteristics of demographic variables.

Variables		Data (n = 300)	
		n	%
Sex	Men	177	59
	Women	123	41
Marital status	Married	272	91
	Single	27	9
Education status	Illiterate	31	10.4
	Elementary	58	19.4
	Middle school	53	17.7
	Diploma	82	27.4
	Academic degree	75	25.1
Employment status	Housewife	102	34.3
	Employed	111	37.4
	Self-employed	84	28.3
Habitation status	Urban	200	66.6
	Rural	100	33.4
See diabetic foot complications in others	Yes	157	52.5
	No	142	47.5
A history of having a diabetic person in the family	Yes	172	57.9
	No	125	42.1
Age of onset of diabetes	≤ 40	60	20
	> 40	240	80
Diabetes duration	≤ 5	107	35.8
	6–10	81	27.1
	> 10	111	37.1

Based on Pearson correlation test results, there was a positive and significant correlation between diabetes health literacy (r = 0.438, p < 0.001), depression (r = − 0.380, p < 0.001), anxiety (r = − 0.355, p < 0.001), stress (r = − 0.423, p < 0.001), and life satisfaction (r = 0.265, p < 0.001) with quality of life. There were negative and significant correlation between diabetes health literacy with depression (r = − 0.372, p < 0.001), anxiety (r = − 0.364, p < 0.001), and stress (r = − 0.370, p < 0.001) (Table 2). Based on the results of linear regression, variables of diabetes health literacy, life satisfaction, self-care behaviors, depression, stress, anxiety, age of onset of diabetes, and diabetes duration were able to predict 31% of QOL (p < 0.001) and diabetes health literacy had the most impact on predicting QOL (p < 0.001) (Table 3).

**Table 2:** Pearson correlation between psychological status, diabetes health literacy, Self-Care, SWLS, and quality of life.

Variables		a	b	c	d	e	f	g	h	i	j	k
Subscales of diabetes health literacy	a. Informational health literacy	1										
	b. Numerate health literacy	0.543*	1									
	c. Communicative health literacy	0.357*	0.337*	1								
d. Diabetes health literacy		0.900*	0.801*	0.595*	1							
Subscales of quality of life	e. Physical health	0.462*	0.232*	0.210*	0.419*	1						
	f. Mental health	0.379*	0.206*	0.127**	0.339*	0.493*	1					
g. Quality of Life		0.486*	0.253*	0.194*	0.438*	0.860*	0.868*	1				
h. Self-care		0.120**	0.172*	0.125**	0.171*	0.054	0.038	0.053	1			
i. SWLS		0.169*	0.059	0.231*	0.182*	0.269*	0.189*	0.265*	− 0.012	1		
j. Depression		− 0.414*	− 0.218*	− 0.157*	− 0.372*	− 0.401*	− 0.258*	− 0.380*	− 0.013	− 0.246*	1	
k. Anxiety		− 0.400*	− 0.205*	− 0.181*	− 0.364*	− 0.386*	− 0.230*	− 0.355*	0.039	− 0.219*	0.874*	1
l. Stress		− 0.395*	− 0.208*	− 0.147*	− 0.370*	− 0.476*	− 0.258*	− 0.423*	− 0.016	− 0.258*	− 0.834*	0.849*

*P < 0.001, **P < 0.05.

**Table 3:** The results of linear regression analysis in predicting quality of life.

Variables	B	SE	Beta	t	P-value	Adjusted R^2^	F	P-value
Diabetes health literacy	0.135	0.034	0.236	4.013	< 0.001	0.31	17.531	< 0.001
Self-care	0.028	0.061	0.023	0.456	0.642			
Life satisfaction (SWLS)	0.138	0.046	0.152	3.010	0.003			
Depression	− 0.123	0.144	− 0.092	− 0.854	0.394			
Anxiety	0.215	0.154	0.157	1.396	0.164			
Stress	− 0.367	0.129	− 0.282	− 2.846	0.005			
Age of onset of diabetes	− 0.041	0.025	− 0.086	− 1.629	0.104			
Diabetes duration	− 0.133	0.040	− 0.181	− 3.351	0.001			

According to Table 4 results, there was a significant relationship between the age of onset of diabetes with stress (p = 0.033) and anxiety (p = 0.017). There was a significant relationship between diabetes duration with the level of depression, stress, and anxiety (p < 0.001) (Table 4). Based on the results of Table 5, there was a significant relationship between diabetes duration with the level of diabetes health literacy (p < 0.001) and subscales of informational health literacy (p < 0.001), numerate health literacy (p = 0.002), and communicative health literacy (p = 0.003) (Table 5). Based on the results of Table 6, there was a significant relationship between diabetes duration and QOL (p < 0.001). There was also a significant relationship between diabetes duration and the self-care behaviors (p = 0.018) (Table 6).

**Table 4:** Relationship between demographic variables and psychological status (depression, stress, anxiety).

Variables		DASS-21 *Mean (SD)*					
		Depression	P-value	Anxiety	P-value	Stress	P-value
Sex^a^	Men	11.42 (3.67)	0.099	11.68 (3.58)	0.205	12.37 (3.77)	0.340
	Women	12.19 (4.13)		12.25 (4.05)		12.82 (4.23)	
Marital status^a^	Married	11.81 (3.81)	0.346	11.99 (3.76)	0.250	12.61 (3.92)	0.363
	Single	11.07 (4.54)		11.11 (4.07)		11.88 (4.50)	
Education status^b^	Illiterate	14.74 (4.34)	< 0.001	14.32 (4.28)	< 0.001	15.58 (3.99)	< 0.001
	Elementary	12.59 (3.91)		12.70 (3.68)		13.10 (3.77)	
	Secondary	12.05 (4.20)		12.16 (3.86)		12.75 (3.93)	
	Diploma	11.18 (3.29)		11.54 (3.45)		12.04 (3.70)	
	Academic	10.24 (3.14)		10.45 (3.21)		11.24 (3.70)	
Employment status^b^	Housewife	12.87 (4.31)	< 0.001	12.91 (4.10)	0.001	13.37 (4.18)	0.002
	Employed	11.62 (3.58)		11.81 (3.62)		12.63 (3.84)	
	Self-employed	10.51 (3.24)		10.80 (3.19)		11.36 (3.56)	
Habitation status^a^	Urban	11.44 (3.65)	0.079	11.64 (3.56)	0.096	12.41 (3.77)	0.440
	Rural	12.34 (4.29)		12.42 (4.18)		12.79 (4.36)	
See diabetic foot complications in others^a^	Yes	11.82 (3.93)	0.744	11.92 (3.91)	0.934	12.57 (4.05)	0.868
	No	11.67 (3.84)		11.89 (3.91)		12.49 (3.86)	
A history of having a diabetic person in the family^a^	Yes	11.82 (3.56)	0.504	12.12 (3.58)	0.251	12.73 (3.76)	0.383
	No	11.56 (3.58)		11.62 (3.91)		12.32 (4.11)	
Age of onset of diabetes^a^	≤ 40	11.03 (3.95)	0.109	10.88 (3.68)	0.017	11.55 (4.07)	0.033
	> 40	11.93 (3.85)		12.17 (3.77)		12.81 (3.90)	
Diabetes duration^b^	≤ 5	10.67 (3.32)	< 0.001	10.72 (3.34)	< 0.001	11.18 (4.09)	< 0.001
	6–10	11.55 (3.88)		11.71 (3.87)		12.48 (4.09)	
	> 10	12.98 (4.06)		13.26 (3.73)		13.99 (3.92)	

^a^Independents sample T-test, ^b^One-way ANOVA.

**Table 5:** Relationship between demographic variables and diabetes health literacy.

Variables		Diabetes health literacy (DHL) *Mean (SD)*							
		Informational health literacy	P-value	Numerate health literacy	P-value	Communicative health literacy	P-value	Total DHL	P-value
Sex^a^	Men	23.01 (4.98)	0.298	10.45 (3.36)	0.498	10.06 (2.24)	0.813	43.53 (8.46)	0.396
	Women	22.33 (5.89)		10.17 (3.76)		10.13 (2.23)		42.63 (9.70)	
Marital status^a^	Married	22.52 (5.36)	0.035	10.30 (3.49)	0.472	10.16 (2.18)	0.053	42.99 (8.95)	0.288
	Single	24.81 (5.21)		10.81 (3.85)		9.29 (2.64)		44.92 (9.48)	
Education status^b^	Illiterate	17.19 (6.21)	< 0.001	7.83 (3.27)	< 0.001	8.09 (2.85)	< 0.001	33.12 (10.40)	< 0.001
	Elementary	19.70 (5.27)		9.56 (3.26)		10.15 (1.79)		39.43 (8.13)	
	Secondary	22.60 (3.95)		9.98 (3.02)		9.96 (2.44)		42.54 (7.59)	
	Diploma	23.86 (4.06)		10.40 (3.09)		10.32 (1.78)		44.59 (6.26)	
	Academic	26.20 (4.11)		12.18 (3.74)		10.68 (2.13)		49.06 (7.57)	
Employment status^b^	Housewife	20.98 (5.38)	< 0.001	9.69 (3.53)	0.005	9.76 (2.33)	0.039	40.44 (9.19)	< 0.001
	Employed	23.88 (5.43)		11.18 (3.46)		10.51 (2.07)		45.58 (9.00)	
	Self-employed	23.32 (4.85)		10.02 (3.40)		9.95 (2.28)		43.29 (7.97)	
Habitation status^a^	Urban	23.57 (4.71)	< 0.001	10.70 (3.54)	0.016	10.24 (2.08)	0.103	44.53 (8.06)	0.001
	Rural	21.04 (6.22)		9.67 (3.41)		9.77 (2.51)		40.48 (10.18)	
Age of onset of diabetes^a^	≤ 40	25.76 (4.10)	< 0.001	12.00 (3.85)	< 0.001	10.40 (2.34)	0.236	48.16 (7.79)	< 0.001
	> 40	21.97 (5.39)		9.92 (3.32)		10.01 (2.20)		41.91 (8.84)	
Diabetes duration^b^	≤ 5	24.99 (4.64)	< 0.001	11.19 (3.77)	0.002	10.45 (2.19)	0.003	46.64 (7.90)	< 0.001
	6–10	22.66 (4.50)		10.28 (3.33)		10.39 (2.10)		43.34 (7.74)	
	> 10	20.57 (5.77)		9.50 (3.21)		9.51 (2.27)		39.59 (9.52)	

^a^Independents sample T-test, ^b^One-way ANOVA.

**Table 6:** Relationship between demographic variables with Self-Care, SWLS, and quality of life.

Variables		Mean (SD)									
		Self-care	P-value	SWLS^c^	P-value	Quality of life (QOL)					
						Physical health	P-value	Mental health	P-value	Total QOL	P-value
Sex^a^	Men	30.55 (4.01)	0.295	22.64 (5.71)	0.531	14.61 (2.92)	0.920	18.10 (3.09)	0.588	32.72 (5.14)	0.707
	Women	31.08 (4.54)		22.22 (5.67)		14.65 (2.97)		18.30 (2.95)		32.95 (5.20)	
Marital status^a^	Married	30.80 (4.07)	0.678	22.56 (5.49)	0.441	14.60 (2.91)	0.508	18.11 (3.05)	0.186	32.72 (5.14)	0.248
	Single	30.44 (5.77)		21.40 (7.47)		15.00 (3.24)		18.92 (2.81)		33.92 (5.35)	
Education status^b^	Illiterate	29.48 (5.16)	0.034	20.61 (5.14)	0.170	12.29 (2.99)	< 0.001	16.48 (2.61)	< 0.001	28.77 (4.88)	< 0.001
	Elementary	32.05 (3.90)		21.96 (5.23)		14.03 (2.66)		17.55 (2.56)		31.58 (4.45)	
	Secondary	30.98 (4.11)		22.05 (4.81)		15.00 (2.71)		18.15 (2.76)		33.15 (4.75)	
	Diploma	30.10 (3.80)		23.23 (5.59)		15.23 (2.82)		18.51 (3.12)		33.74 (5.08)	
	Academic	30.80 (4.37)		23.10 (6.75)		15.20 (2.87)		19.06 (3.28)		34.26 (5.18)	
Employment status^b^	Housewife	31.35 (4.54)	0.236	21.50 (4.99)	0.065	14.28 (2.86)	0.029	18.11 (2.82)	0.086	32.40 (5.03)	0.023
	Employed	30.42 (4.02)		22.61 (6.29)		14.37 (3.05)		17.79 (3.27)		32.17 (5.35)	
	Self-employed	30.54 (4.12)		23.43 (5.42)		15.33 (2.78)		18.76 (2.92)		34.09 (4.90)	
Habitation status^a^	Urban	30.39 (4.11)	0.034	21.91 (5.57)	0.265	14.78 (2.89)	0.243	18.28 (3.13)	0.395	33.07 (5.14)	0.244
	Rural	31.50 (4.45)		22.70 (5.75)		14.36 (3.06)		17.97 (2.85)		32.33 (5.24)	
Age of onset of diabetes^a^	≤ 40	30.71 (4.16)	0.908	21.56 (6.05)	0.167	15.51 (3.13)	0.009	18.91 (3.28)	0.037	34.43 (5.61)	0.006
	> 40	30.78 (4.26)		22.70 (5.58)		14.40 (2.85)		18.00 (2.94)		32.41 (4.97)	
Diabetes duration^b^	≤ 5	29.84 (4.84)	0.018	22.85 (6.45)	0.629	15.57 (3.00)	< 0.001	18.98 (3.31)	0.001	34.56 (5.39)	< 0.001
	6–10	31.40 (3.81)		22.39 (5.38)		15.02 (2.58)		18.12 (2.76)		33.14 (4.29)	
	> 10	31.17 (3.76)		22.45 (5.69)		13.38 (2.69)		17.47 (2.77)		30.86 (4.89)	

^a^Independents sample T-test, ^b^One-way ANOVA, ^c^Satisfaction with Life Scale (SWLS).

## Discussion

This study is a cross-sectional study was aimed to determine the role of diabetes health literacy, psychological status, self-care behaviors, and life satisfaction in predicting QOL in Iranian patients with type 2 diabetes. The results of this study showed that there was a positive and significant correlation between health literacy and QOL. Also, diabetes health literacy, life satisfaction, self-care behaviors, depression, stress, anxiety, age of onset of diabetes, and duration of diabetes predict 31% of QOL in type 2 diabetes patients and diabetes health literacy has the greatest impact.

The results of this study showed that there was a significant relationship between education status and depression, but this relationship was reversed in our study. This inverse relationship seen in our study may be due to people's awareness of the disease, and this itself can reduce depression, stress and anxiety in a person. In addition, a person with more knowledge is likely to take better. The study of Sweileh indicated that people who had more education were doing better self -care behavior^47^.The study by Zendegani showed no significant relationship between education status and depression, which may indicated that patients with high education status do not understand the important of self-care behaviors^48^. In our study, may be people with higher education status have more health literacy than diabetes, with increasing education status they experience less depression, stress, and anxiety, but in the previous study, may be this association of education and health literacy has not occurred.

In this study, there was a significant relationship between the duration of the disease and depression, and the severity of depression increased with the duration of the disease. These results are consistent with the results of the study of Mosaku et al.^49^, whereas in the study by Zendegani^48^ the incidence of depression decreased with increasing duration of diabetes. The increase in depression among patients in this study could be due to persistent self-care behaviors, diabetes burnout, or fear of possible diabetic complications.

The results of this study also showed that there was a relationship between education status and the health literacy of diabetes, which is consistent to the results of other studies^50–52^. Usually, with increasing levels of education, people are likely to increase health literacy in different aspects of the disease, which can have a positive effect on self -care behaviors, and this makes them more effective in health follow-up and interaction with their caregivers. to perform better and because of their relative knowledge, they better understand the reason for follow-ups and compliance with treatment orders. As a result, this enables them to conduct health follow-ups and interactions with caregivers more effectively, they better understand the reasons for follow-ups and compliance with treatment orders.

There was a significant relationship between work status and diabetes health literacy, with employed individuals showing higher levels of diabetes health literacy, which is consistent with the findings of Noroozi^50^. This problem may be due to the people interaction with the environment outside the home and with different people, which can improve their health literacy levels. Contrary to the results of this study, results of several studies showed a direct relationship between the duration of diabetes and health literacy^53,54^. It is usually expected with increase duration period of the disease, the level of health literacy and the experiences of diabetes management increase, but in our study, a contradictory result was obtained. It seems that it may be the lack of awareness about the long-term complications of the disease, or another reason may be the decrease in people's sensitivity to the disease and the lack of updating their information by health care providers, which has decreased people's health literacy with the increase in the duration of diabetes. Therefore, in order to improve the health literacy level of patients, it is necessary to design and implement programs such as education classes for people with low education and older age. According to the results of this study, there was a significant relationship between education status and self-care behaviors, which is consistent with the findings of other studies^55–57^. Given that more than 50% of patients in this study had diplomas and higher education, most self-care behaviors were justified.

There was also a significant relationship between the duration of the disease and the self -care behaviors, and with the prolongation of the disease duration, the self-care behavior increases, which was in line with the results of other studies^58,59^. Dietary adherence and better and sustainable relationships with physicians and healthcare providers may be among the reasons for improved self-care behaviors as diabetes duration increases.

The results of this study showed that there was a significant relationship between education status and QOL, and with the increase in the level of education, QOL of patients increased, which was consistent with the results of Glasgow et al.^60^. Higher QOL among patients with increased educational attainment may be due to increased levels of health literacy and better self-care behaviors.

Also, there was a significant relationship between the duration of diabetes and QOL, and with the increased duration of the disease, QOL was reduced in patients, which was consistent with the results of other studies^61,62^. Reducing QOL can be due to numerous complications of diabetes as a result of prolonging the disease.

Stress was also one of the predictors of QOL, and QOL decreased as stress score increased, which is consistent with other studies^12,63^. In the interpretation of this finding, it can be argued that the fear of the problems and consequences of diabetes and the inability to solve these problems can increase stress in patients and thus reduce their QOL. Stress can also lead to lack of dietary adherence, reduced physical activity, and smoking in diabetic, resulting in reduced self-care behaviors and ultimately lower QOL^64^.

Another predictor of patients' QOL in this study was their diabetes health literacy level, meaning that as their health literacy level improved, their QOL improved. Results from similar studies have shown that health literacy is one of the effective factors in improving self-care behaviors, glycemic control (HbA1c) and improving QOL in type 2 patients^65^. Another study found that improving health literacy can increase self-care behaviors and improve QOL in people with diabetes^66^. It seems that health literacy makes people with diabetes pay more attention to self-care, they will be more obedient to the orders of doctors and health care providers, and they are likely to experience fewer complications of the disease; this makes people with type 2 diabetes have a higher QOL.

In this study, life satisfaction was also an effective factor in predicting patients' QOL. With the improvement of life satisfaction, QOL also improved. Given that there was a negative and significant correlation between stress, anxiety and depression with life satisfaction, it seems that reducing these problems can make patients more satisfied and thus increase their QOL. The results of one study showed that patients who are satisfied with the treatment of their illness have a higher QOL^67^. In fact, the more satisfied people are with their lives, the less stress, anxiety, and depression they experience, and ultimately, they can experience a higher QOL.

Finally, the models in our study showed the large impact of diabetes health literacy on QOL. Health literacy appears to be effective for all psychological factors in people with diabetes, and improving health literacy in these patients can reduce their depression, anxiety and stress and improve their QOL. Informing type 2 diabetes about their disease in such a way that they know their disease and know its complications and eventually leads to people's compliance with the treatment staff's orders can provide a better health status and a higher QOL for patients.

## Conclusion

The results of this study showed that diabetes health literacy, life satisfaction, stress and duration of diabetes can predict QOL of diabetic, and our study highlights the impact of health literacy on QOL in diabetic. Therefore, it is necessary to pay more attention to these effective variables when planning educational programs and design appropriate programs for this field. In fact, by increasing diabetes health literacy of type 2 diabetic, we can maintain and improve their QOL, because increasing diabetes health literacy level of people makes the patients aware of their disease, act more committed to medical orders, and have more self-care, and finally, they will suffer less stress, anxiety, or depression. In fact, these mental complications can cause patients to fall into a vicious circle. The more stress, anxiety, and depression a person has, the worse the diabetes will be, and the worse the mental state will be. Therefore, increasing the level of health literacy can reduce many of these cases and provide a better quality of life for patients.

## Data Availability

All data generated or analysed during this study are included in this published article.

## Abbreviations

SWLS: Satisfaction with life scale
DASS-21: Depression, anxiety, and stress questionnaire
SF-12: Quality of life questionnaire
QOL: Quality of life
SD: Standard deviation
DHL: Diabetes health literacy
